# A protein with broad functions: damage-specific DNA-binding protein 2

**DOI:** 10.1007/s11033-022-07963-4

**Published:** 2022-10-03

**Authors:** Ning Bao, Jiguang Han, Huimin Zhou

**Affiliations:** 1 Harbin Medical University Cancer Hospital, Harbin, Heilongjiang China; 2 Breast Plastic Surgery Department, Harbin Medical University Cancer Hospital, 150 Haping Road, Nangang District, Harbin, 150000 China

**Keywords:** Damage-specific DNA-binding protein 2 (DDB2), Nucleotide excision repair (NER), Apoptosis, Premature senescence, Cancer, Chemoradiotherapy

## Abstract

Damage-specific DNA-binding protein 2 (DDB2) was initially identified as a component of the damage-specific DNA-binding heterodimeric complex, which cooperates with other proteins to repair UV-induced DNA damage. DDB2 is involved in the occurrence and development of cancer by affecting nucleotide excision repair (NER), cell apoptosis, and premature senescence. DDB2 also affects the sensitivity of cancer cells to radiotherapy and chemotherapy. In addition, a recent study found that DDB2 is a pathogenic gene for hepatitis and encephalitis. In recent years, there have been few relevant literature reports on DDB2, so there is still room for further research about it. In this paper, the molecular mechanisms of different biological processes involving DDB2 are reviewed in detail to provide theoretical support for research on drugs that can target DDB2.

## Introduction

DDB2 (damage-specific DNA-binding protein 2, also known as the p48 subunit) is a DNA repair nuclear protein encoded by the XPE gene [1–3]. It was originally identified as a component of the human damage-specific DNA-binding heterodimeric complex UV-DDB, which is involved in the early stage of ultraviolet radiation (UV)-induced nucleotide excision repair (NER) [4, 5]. DDB2 interacts with DDB1 (damage-specific DNA-binding protein 1), similar to the CSA (Cockayne-syndrome A) protein [6]. UV-DDB forms a larger complex through the binding of the DDB1 linker to the cullin 4A (CUL4A)–regulator of cullin 1 (ROC1) E3 ubiquitin ligase (CRL) complex [7]. DDB2 acts as a substrate receptor in this structure, providing substrate specificity capable of recognizing lesions induced by UV radiation [8]. DDB2 is ubiquitously expressed in human tissues, with a higher expression in the testis, kidney, liver, and corneal endothelial cells and lower expression in the brain, lung, heart, skin, and muscle [9]. Homologs of human DDB2 are only found in mammalian species; the mouse DDB2 homolog is only 73% identical in amino acid sequence compared to human DDB2 [9]. The human DDB2 gene is located on chromosome 11p12-p11, with a molecular weight of 48 kDa, and encodes a protein composed of 428 amino acids [4, 9]. The DDB2 protein contains a terminal seven-bladed WD40 β-propeller domain (residues 137–454), disordered N-terminal tail, and a helix-loop-helix motif (residues 101–136) [10]. The WD40 domain mediates interactions between the protein and DNA, while the N-terminal helix-loop-helix fragment of DDB2 interacts with the two short repeating β-propeller domains of DDB1, which are essential for the biological function of DDB2 [2, 10]. P53 can bind to the 5′-sequence of DDB2 and activate gene expression [11]. The P53 protein interacts with BRCA1 (breast cancer-associated protein 1) to enhance the binding of p53 to the DDB2 promoter and induce DDB2 activation [11, 12]. In addition, TAp63γ (tumor protein 63 isoform gamma), which is structurally similar to p53, can also activate DDB2 expression by identifying the same region upstream of the transcription start site [13]. Interestingly, DDB2 can also directly regulate the expression of p53 through a positive feedback loop [14]. Notably, the expression of DDB2 is not entirely dependent on p53, as other mechanisms can increase its expression in p53^−/−^ cells [15]. Roy et al. confirmed that ROS could activate the expression of DDB2 in a p53-independent manner [1]. In addition, ROS is related to the activation of transcription factor AP1 by the p38MAPK/JNK pathway, which enhances the binding of AP1 to the DDB2 promoter, thereby increasing DDB2 transcription [1].

The level of DDB2 gradually increases in the mid-G1-phase, peaks at the G1/S boundary, and then declines in the S phase [16]. An analysis of the DDB2 transcriptional regulatory regions revealed core promoter regions (located within 220 bp upstream of the transcription start site) associated with cell cycle-regulated genes: no TATA box, a G/C-rich region, an NF-1 element, and four Sp1 elements [17]. The Sp1#1 element (+ 29 to − 22) is closest to the transcription start site and is a crucial determinant of promoter activity [17]. Mutations in this element resulted in a significant reduction in transcriptional activity to 17% [17]. Additionally, an E2F element was identified downstream of the DDB2 transcription start site [9, 17]. After mutating the E2F sequence (+ 36 to + 43), the promoter activity was reduced to 74%, which had a negligible effect on the regulation of DDB2 [17]. DDB2 stimulates the transcriptional activity of the transcription factor E2F1, thereby stimulating the expression of multiple target genes involved in cell cycle progression [16, 18]. DDB-2 mediated cell cycle regulation partially involves post-transcriptional mechanisms. The internal ribosome entry site (IRES) element at the 5′-end of the DDB2 mRNA and the uracil-rich sequence in the 3′-untranslated region play important roles in DDB2 translation [2, 19]. Further studies revealed that the Cul4A (Cullin 4A) protein induces the proteolysis of DDB2 through its ubiquitin ligase function during DNA repair [16, 20]. This process is also associated with the COP9 signalosome [6]. Other studies have found that DDB2 degrades the cell cycle-dependent kinase (CDK) inhibitors p27 and CDT2 [21, 22]. Recent mechanistic studies have revealed that DDB2 also stabilizes CDT1 by degrading CDT2 in a PCNA-independent manner and promotes the recruitment of MCM and assembly of pre-RC to ensure that DNA replication is initiated in the S phase [22].

This article explored the roles of DDB2 in biological processes that may affect cancer, including NER, apoptosis, and premature senescence. We then summarized multiple recent studies describing the regulatory role of DDB2 as an oncogene or tumor suppressor gene in different cancers, as well as its influence on cancer sensitivity to radiotherapy and chemotherapy. In addition, new roles for DDB2 in other diseases, including hepatitis, are also discussed. This study aims to provide new targets for treating various diseases, including cancer, and provide theoretical support for further research on DDB2.

## The role of DDB2 in NER

NER consists of two distinct sub-pathways, global genome NER (GG-NER) and transcription-coupled NER (TC-NER), which are the main pathways used to remove massive DNA damage in mammals [23]. GG-NER repairs DNA damage both in transcribed and non-transcribed DNA strands. The XPC-RAD23B complex affects aberration recognition [24], and DDB2 specifically binds to the most common UV-damaged DNA damage, including (6–4) pyrimidine-pyrimidine ketone photoproducts (6-4PPs) and cyclobutane pyrimidine dimers (CPDs), especially critical for the repair of the latter [10, 25]. In addition, DDB2 binds to various other forms of damaged DNA, including 8-oxoguanine (8-oxoG), abasic sites, and single-stranded DNA, and the damage products induced by cisplatin, nitrogen mustard, and psoralen [4, 26]. In contrast, TC-NER occurs only in transcribed DNA strands, and RNA polymerase II affects the initial damage recognition instead of XPC-RAD23B [9, 24].

### Ubiquitination of DDB2

DDB2 is involved in GG-NER [10]. Wakasugi et al. used a combination of micropore UV irradiation and immunostaining and found that FLAG-tagged p48 (DDB2) was transferred to the UV-irradiated area immediately after irradiation, suggesting that DDB2 participates in the early steps of DNA repair [27]. Many NER models are known to involve the protein ubiquitination function of DDB2. An initial report indicated that DDB2 utilizes its nuclear import function to recruit DDB1 into the nucleus and recognize UV-induced DNA damage for DNA repair [3]. After damage recognition, the Cul4A-DDB1-DDB2 complex recruits XPC to the damage site and ubiquitinates DDB2 [28, 29]. The polyubiquitination of DDB2 results in a decrease in its affinity for DNA damage [28]; hence, DDB2 is stimulated to extract from DNA by the ubiquitin-dependent segregase p97/VCP, followed by proteasome-mediated degradation [30, 31]. During this process, the ubiquitination proportion of XPC decreases but eventually stabilizes, and its binding ability to DNA is enhanced [28, 32]. XPC can relocate from internucleosomal DNA fragments to untreated residual UV lesions within the nucleosome core particles [33]. Other studies have shown that the Cul4A-DDB1-DDB2 complex also monoubiquitinates histones H2A25, H3, and H4 at DNA lesion sites and promotes histone removal from nucleosomes at the damage site, which contributes to chromatin dedensification to help repair factors, such as XPC, enter lesions [34–36].

### The SUMOylation and PARylation of DDB2

The post-translational modification of proteins mediated by small ubiquitin-related modifiers (SUMOs) is critical for maintaining genome stability [37]. SUMOs are covalently linked to proteins through ubiquitin-like enzymatic cascade activities. A co-immunoprecipitation study demonstrated that DDB2 was SUMOylated by the SUMO E3 ligase PIASy (a protein inhibitor that activates the STST protein) after UV damage [38]. Further screening of the DDB2 sequence revealed three potential SUMO modification sites by mutating lysine residues (Lys5, Lys77, and Lys309) to arginine (Arg) [39]. Under UV irradiation, mutations in Lys309 completely abolished DDB2 modification, whereas mutations in Lys5 and Lys77 did not affect UV-induced DDB2 modification, suggesting that SUMOylation at Lys309 is functionally significant [39]. This modification is necessary to recruit XPC to DNA damage sites and regulate the efficient repair of CPDs.

In addition, it has been reported that DDB2 is PARylated in a PARP-1-dependent manner, which stabilizes UV-DDB at the damaged site [40]. Shah et al. used immunoblotting to reveal that PARP inhibitors prevent the interaction of DDB2 with PARP1 or XPC [41]. Further studies have found that a lack of PARP-1-mediated DDB2 PARylation results in the delayed recruitment of XPC [41]. In summary, ubiquitination, PARylation, and SUMOylation are important post-translational modifications (PTMs) that influence the NER process. Other studies have speculated that DDB2 may be involved in NER through chromatin remodeling at DNA damage sites. It has been reported that DDB2 is involved in the activation of the SWI/SNF (SWItch/sucrose non-fermenting) chromatin remodeling complex and the CREB (cAMP-response-element-binding protein)-binding protein (CBP)/P300 histone acetyltransferase complex. These processes play an important role in transcription, replication, and DNA repair [42, 43].

## DDB2 mediates apoptosis and premature senescence

In addition to NER, DDB2 also contributes to cell apoptosis and senescence. Mouse embryonic fibroblasts (MEFs) and human cells lacking DDB2 expression are resistant to apoptosis induced by DNA damage caused by UV irradiation and chemotherapeutic drugs [44, 45]. P21 Waf1/Cip, a cyclin-dependent kinase (CDK) inhibitor, acts as a barrier against apoptosis by interacting with the pro-apoptotic molecules ASK1, procaspase 3, and caspase 8 [46]. In the case of DDB2 depletion, p21 Waf1/Cip1 accumulates in cells, blocks apoptosis, and causes S-phase arrest. This notion can be verified by the efficient apoptosis observed in DDB2^−/−^p21^−/−^ mice [47]. Thus, DDB2 ensures efficient apoptosis by downregulating p21 expression in DNA-damaged cells. Furthermore, apoptosis may be associated with DDB2-induced CDT2 degradation in cancer cells [22].

High levels of p21Waf1/Cip1 were also associated with enhanced senescence responses; however, DDB2-deficient cells remained resistant to oxidative stress, oncogenic effects, and DNA damage-induced senescence even with high levels of p21 Waf1/Cip1 [45, 48]. Another study found that MEFs isolated from DDB2^−/−^ embryos lacked p19Arf, a key factor in senescence induction. P19Arf overexpression rescues the phenotype of senescence-deficient cells [48]. Some studies have shown that reducing the expression of DDB2 can inhibit the Ras-mediated premature senescence response [45]. These results indicate that DDB2 is an essential mediator of premature senescence. Further studies have revealed a positive feedback process between DDB2 and reactive oxygen species (ROS). ROS stimulates the expression of DDB2, then DDB2 restricts the expression of antioxidant genes by modifying the chromatin of MnSOD and catalase. This chromatin modification leads to the continuous accumulation of ROS, resulting in premature senescence [1, 45, 47]. Interestingly, the increased accumulation of ROS caused by high levels of DDB2 is detrimental to the age-related pathophysiology in the elderly population. Single nucleotide polymorphisms (SNPs) of the DDB2 gene have been reported to be associated with aging-related degenerative diseases such as arteriosclerosis and neurodegenerative disease progressive supranuclear palsy [49, 50].

## DDB2 and cancer

**Fig. 1:**
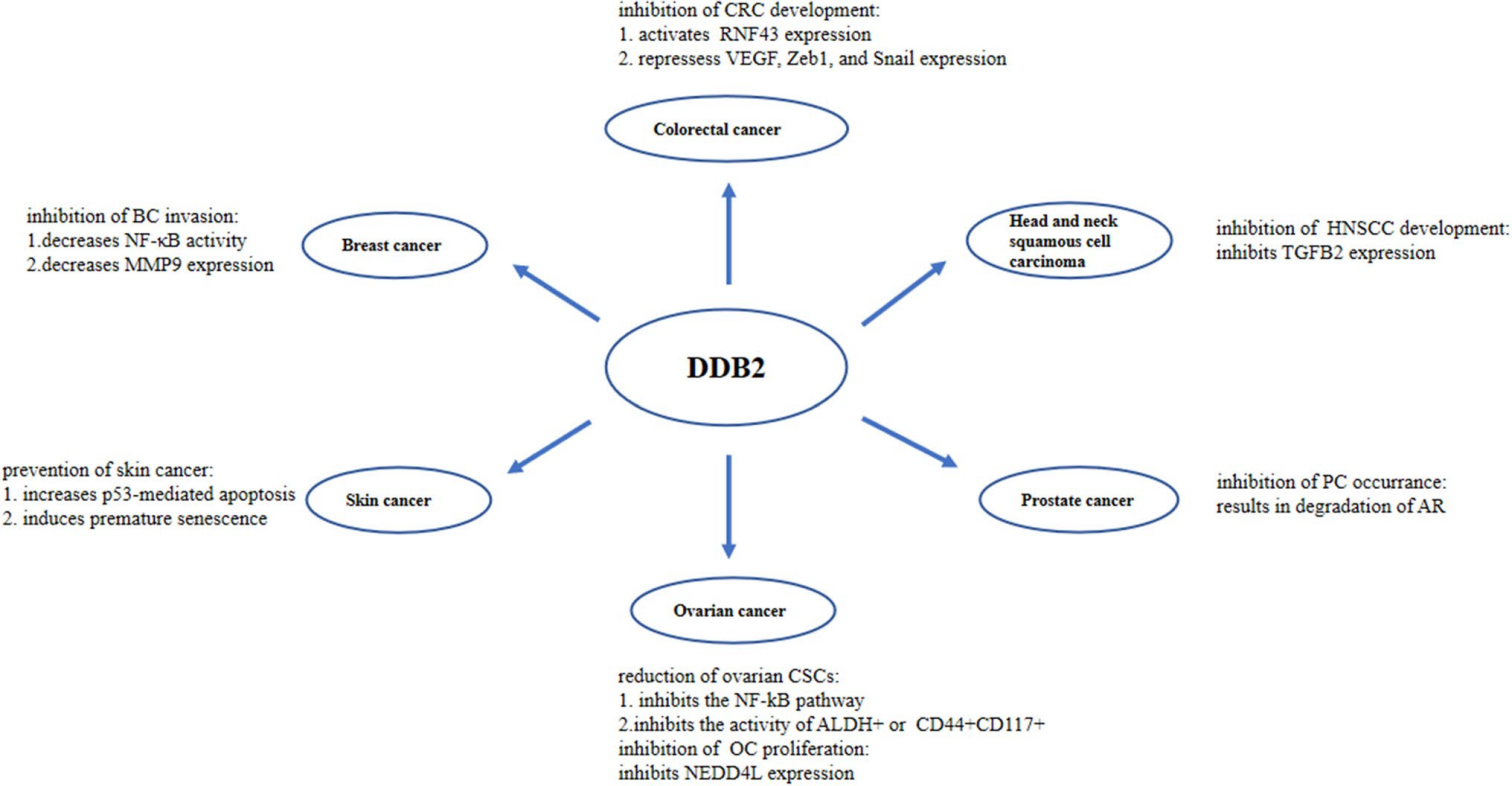
Antitumor mechanism of DDB2 in different cancers

**Fig. 2:**
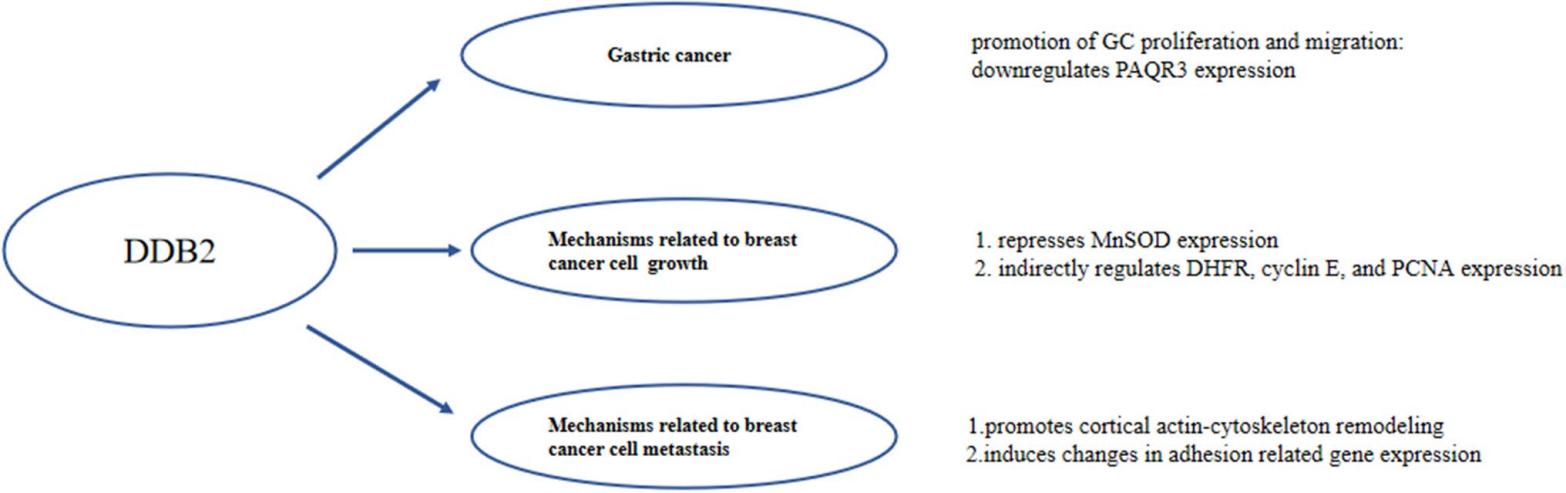
Cancer promoting mechanism of DDB2

DDB2 may also target other proteins for degradation during tumor progression and affect several stages of carcinogenesis, such as cancer cell proliferation, survival, epithelial-to-mesenchymal transition, migration and invasion, angiogenesis, and cancer stem cell formation. The specific mechanisms depend on the type of tumor involved. In this section, we focused on the biological effects and potential molecular mechanisms of DDB2 on the occurrence and development of various cancers. Figures 1 and 2 summarize the roles of DDB2 in different cancers.

### XPE and skin cancer

Xeroderma pigmentosum (XP) is a rare autosomal recessive inherited disorder associated with defects in the NER protein [51]. XP consists of eight complementary groups (XPA, XPB, XPC, XPD, XPE, XPF, XPG, and XPV) [52]. Among them, the number of patients with xeroderma pigmentosum group E (XPE) is low; currently, approximately 20 cases have been reported in the literature. Among patients with XP, those with XPE have the lowest sensitivity to sunlight. Compared to patients with XP in the other complement groups, patients with XPE are 1,000 times more likely to develop skin cancer [4, 52]. This type of disease is significantly associated with single amino acid substitutions or C-terminal truncations induced by DDB2 mutations [53, 54]. More than ten types of DDB2 mutations are known, among which substitution mutations are the most common. The most common of these substitution mutations are found in exon 7 of the DDB2 gene (refer to Table 1 for further detail) [51–60]. Ebru found that changes in the shape, distribution, and quantity of argyrophilic nucleolar organizing region-associated protein (AgNOR) can provide information about the diagnosis and prognosis of patients with XPE, thereby guiding physicians regarding effective treatment strategies [61].

**Table 1:** Summary of DDB2 mutations in XPE patients

Date	Patient	Origin	Nucleotide (Exon)	Amino Acid	Zygosity	References
1970	34F XP2RO	Netherlands	c.818 G>A (Ex 6)	p. Arg273His	Homozygous	[55]
1970	29F XP3RO	Netherlands	c.818 G>A (Ex 6)	p. Arg273His	Homozygous	[55]
1996	41F XP82TO	Japan	c.730 A>G (Ex 6)	p. Lys244Glu	Homozygous	[53]
1999	62F Ops1	Japan	c.937 C>T (Ex 7)	p. Arg313X	Homozygous	[54]
2003	21F GM01389	USA	c.1049 T>C (Ex 8)/c.1045_1047del (Ex 8)	p. Leu350Pro/p. Asn349del	Heterozygous/Heterozygous	[56]
2003	18M XP23PV	Italy	c.703_1023del (del. Ex 6 and 7)	p. Leu235_Lys341del	Homozygous	[56]
	29F XP25PV		c.919 G>T (Ex 7)/c.918 G>A (Ex 7)	p. Asp307Tyr/No change	Homozygous/Homozygous	[56]
	35F XP27PV		c.730_733del (Ex 6)/c.703_880del. (Ex 6)/c.703_1023del (Ex 6 and 7)	p. Lys244X/p.Trp236Valfs*10/p. Leu235_Lys341del	Homozygous/Homozygous/Homozygous	[56]
2007	A 12-year-old boy	Finland	c.574 C>T (Ex 3)	p. Arg192X	Homozygous	[57]
2011	45F XP37BE	USA	c.818 G>A (Ex 6)	p. Arg273His	Homozygous	[58]
2011	43M XP66BE	USA	c.818 G>A (Ex 6)	p. Arg273His	Homozygous	[58]
2011	45M XP1GO	German	c.914 C>A (Ex 7)	p. Thr305Asn	Homozygous	[58]
2011	53F XP408BE	USA	c.1049 T>C (Ex 8)	p. Leu350Pro		[58]
2016	49F XP105BR	Caucasian	c.1070 C>T (Ex 7)/c.716 G>T (Ex 4)	p. Pro357Leu/p. Arg239Ile	Heterozygous/Heterozygous	[51]
2016	62F XP98BR	Caucasian	c.161 G>A (Ex 1)	p. Trp54X	Homozygous	[51]
2016	62F XP100BR	Caucasian	c.457–2 A>C (Ex 3)	Splice	Homozygous	[51]
2016	30F XP115BR	Pakistani	c.1149delG(Ex 7)	p. Met383fs	Homozygous	[51]
2017	XP51-MAH-1	Tunisian	c.1138delG(Ex 7)	p. Lys381Argfs*2	Homozygous	[59]
2020	18F, 5F, 11F	Iraqi	c.1063 C>T (Ex 8)	p. Arg355Ter	Homozygous	[52]
2020	28F	China	c.111_112del(Ex 1)	p. A39Efs*6	Homozygous	[60]

Several studies have reported that the absence of DDB2 increases the risk of tumor formation in mice after UV induction. Yoon et al. found that DDB2-knockout mice are more prone to skin cancer after UV exposure than wild-type mice. DDB2-knockout mice also had a higher chance of developing spontaneous tumors, consistent with previous observations [44, 62]. Alekseev et al. studied the transgenic mouse line K14-DDB2 (mice that ectopically express mouse DDB2 in their epidermal cells) and found that a high expression of DDB2 improved the repair of photodamage products in skin fibroblasts, delaying and reducing the occurrence of skin squamous cell carcinoma, and increased tumor-free survival [20]. Recent mechanistic studies have shown that DDB2 prevents UV-induced skin cancer by increasing p53-mediated apoptosis of UV-damaged cells and maintaining high levels of ROS to induce premature senescence. METTL14 was also found to promote m6A (N6-methyladenosine) and YTHDF1 (an m6A reader that promotes translation of m6A-modified transcripts)-mediated DDB2 translation, which promotes GGR to inhibit ultraviolet B (UVB) radiation-induced skin carcinogenesis [44, 45, 63]. These results suggest the protective role of DDB2 against skin tumor formation.

### Colorectal cancer (CRC)

The Wnt/β-catenin signaling pathway is crucial in colon carcinogenesis [64]. Tumor staining in DDB2-knockout mice showed that the expression of the Wnt signaling inhibitor RNF43 (ring finger protein 43) decreased and the mRNA expression of the Wnt target gene Cdx1 increased [64, 65]. Mechanistic research indicated that DDB2 recruits β-catenin to the upstream P2/P3 regulatory region of RNF43 to activate RNF43 by interacting with the H3K27 methylase EZH2 (zeste homolog enhancer 2) [64, 66]. DDB2 can also activate the expression of RNF43 by regulating the interaction of the upstream regulatory region of RNF43 with its TCF4-binding region, which removes the Wnt receptor FZD5 from the cell surface and ultimately downregulates Wnt signaling in CRC cells [64]. These findings show that DDB2 inhibits colon cancer development by suppressing the Wnt signaling pathway.

Recently, Yang et al. detected 300 CRC, 300 adjacent, and 214 normal tissues and found that the protein expression of DDB2 in CRC tissues was higher than in non-tumor and adjacent tissues [67]. In addition, low DDB2 expression was found in colon cancer of the T3-T4 stage and infiltrative growth type, similar to the tendency of decreased DDB2 expression in high-grade colon cancer found by Roy et al.[68] Survival analysis showed that high DDB2 expression was associated with good survival in colorectal cancer patients (adjusted HR 0.20, 95% CI 0.06–0.72, P = 0.014) and female colorectal cancer patients (adjusted HR 0.27, 95% CI 0.08–0.92, P = 0.036) [67]. Epithelial-mesenchymal transition (EMT) is fundamental to the malignant progression of cancer [69]. Roy et al. found that DDB2-knockout mice had a higher incidence of lung and liver metastases [68]. The loss of DDB2 promotes the development of metastatic colon cancer and transformation to a mesenchymal phenotype, increasing the expression of mesenchymal markers N-cadherin and vimentin and decreasing the expression of E-cadherin. DDB2 can also lead to the trimethylation of VEGF, Zeb1, and Snail promoter histone H3K9 by recruiting the histone methyltransferase Suv39h and transcriptionally repressing the expression of these EMT-inducing genes [68]. However, in metastatic colon cancer, DDB2 is repressed by the overexpression of miR-675-5p, allowing EMT-related gene expression [70]. Therefore, we speculate that miR-675-5p is expected to become a potential new target for the treatment of metastatic colon cancer. In addition, the primary function of DDB2 in regulating EMT is conserved in different types of tumors; hence, DDB2 expression in tumors may serve as a predictor of EMT progression [68, 71].

### Gastric cancer (GC)

Machlowska et al. performed high-throughput sequencing in patients with GC and revealed that DDB2 (rs326212) contributes to gastric carcinogenesis [72]. Through colony formation and MTT assays, we found that the proliferation ability of DDB2-knockout GC cells was significantly decreased [73]. In addition, the results of Transwell and wound-healing assays showed that the migration ability of cells in the DDB2-knockout group was significantly limited [73]. In contrast, the knockout of PAQR3 (progestin and adipoQ 3) rescued the effects of DDB2 deletion on cell proliferation and migration.[73] As a newly discovered tumor suppressor, the function of PAQR3 is mainly due to its negative modulatory roles in Raf/MEK/ERK signaling pathway [74]. The N-terminal amino acid residues 40–60 of the PAQR3 receptor interact with the WD domain of DDB2. DDB2 targets Lys61 of PAQR3 for ubiquitination and degradation, which alters its protein expression level [73]. In addition, DDB2 alters the regulation of PAQR3 on EGF (epidermal growth factor (EGF) and insulin-initiated cell signaling [73]. These results suggest that DDB2 plays an active role in gastric carcinogenesis by regulating PAQR3 expression. In addition, detection of NER pathway polymorphisms, such as in DDB2 and XPC, may be applied to future GC risk prediction algorithms to provide personalized prevention strategies for GC [75].

### Head and neck squamous cell carcinoma (HNSCC)

Hypoxia activates the expression of genes involved in EMT and metabolic reprogramming, including ZEB1, TWIST, SNAIL, and VEGF, in multiple cancers by stabilizing and inducing hypoxia-inducible transcription factors (HIFs) [76, 77]. DDB2 is upregulated under hypoxic conditions. A Kaplan–Meier analysis of data from 81 patients with HNSCC from a publicly available dataset (Oncomine) showed that patients with higher DDB2 expression had longer survival than those with lower DDB2 expression, which was significant at an alpha level of 0.05 (log-rank P = 0.0404) [71]. Bommi et al. found that DDB2 regulates multiple hypoxic signaling genes by downregulating HIF1α expression in HNSCC, preferentially inhibiting the expression of TGFB2 mRNA (transforming growth factor B2 mRNA), the primary regulator of Snail and Zeb1 [78]. The DDB2 homology element in the HIF1A promoter matched (90%) with DDB2-binding core sequences in the MnSOD and BCL2 promoters [79, 80]. Furthermore, the epigenetic regulation patterns of the HIF1A promoter by DDB2 and Suv39h1 via histone-marked H3K9Me3 were similar to the constitutive repression of DDB2-mediated EMT-related regulators in colon adenocarcinoma cells [68]. These results highlight the antitumor effect of DDB2 and suggest its therapeutic value in HNSCC.

### Ovarian cancer (OC)

Cancer stem cells (CSCs) are the most important trigger for the occurrence and progression of many cancers, including OC [81]. Several studies have shown that DDB2 inhibits the NF-kB pathway and the activity of stem cell markers, acetaldehyde dehydrogenase (ALDH+) or CD44+CD117+, to reduce the abundance of ovarian CSCs, elucidating a new mechanism for the DDB2-induced inhibition of cancer cell tumorigenicity [82]. Han et al. found that the inhibition of the NF-κB pathway is partly due to the DDB2-induced upregulation of IκBα expression (an inhibitor of NF-κB) in OC cells and ovarian CSCs, which inhibits the self-renewal ability of ovarian CSCs and eventually leads to tumor progression disorder. The results of Ennen et al. are consistent with this finding [82, 83]. Another study found that DDB2 binding to the ALDH1A1 promoter leads to the enrichment of histone H3K27me3 or directly competes with the transcription factor C/EBPβ, inhibiting ALDH1A1 promoter activity during the process [84]. Cui et al. suggested that DDB2 abrogates ovarian CSC properties and inhibits dedifferentiation by downregulating ALDH1A1 expression [84]. Neural progenitor cell expression developmental downregulated gene 4-Like (NEDD4L) is a newly discovered target gene of DDB2 [85]. High expression of NEDD4L is associated with poor prognosis in patients with advanced OC [85]. It can inhibit transforming growth factor signaling through the targeted activation of Smad2/Smad3 degradation [85]. In addition, the study demonstrated that DDB2 also enhances transforming growth factor-β (TGF-β) signaling by inhibiting the transcription of NEDD4L, showing antiproliferative properties in an in vitro OC model [86]. Here, the TGF-β-induced phosphorylation of Smad2 is significantly increased, and cells respond to TGF-β-induced cell growth inhibition. Therefore, DDB2 plays a protective role as a tumor suppressor gene in the onset and development of OC.

### Prostate cancer (PC)

A recent study found higher expression levels of nuclear receptor-interacting protein (NRIP) and androgen receptor (AR) and lower DDB2 expression in PC tissues than in non-tumor tissues. NRIP is an androgen-regulated gene belonging to the CUL4-DDB1-binding protein family (DCAFs), such as DDB2 [87]. Chang et al. demonstrated that DDB2 interacts with the AR, resulting in its ubiquitination and proteasomal degradation. NRIP competes with DDB2 for binding to the HBD (C-terminal hormone-binding domain) of AR in the AR-DDB2-DDB1-CUL4A complex, protecting the AR [87]. Several studies have demonstrated that PC is closely associated with AR [88, 89]. As the expression level of DDB2 in PC tissues is lower than in non-tumor tissues, the balance between NRIP and DDB2 is disrupted. The homeostasis of the AR is then disturbed, which may trigger AR-dependent PC. These studies suggest DDB2 as a potential therapeutic target for PC.

### Breast cancer (BC)

It has been reported that a low expression of DDB2 promotes the invasion and metastasis of BC [83]. Relevant mechanistic studies have shown that DDB2 decreases NF-κB activity and the expression of matrix metalloproteinase 9 (MMP9) by upregulating IκBα gene expression, limiting cancer cell invasiveness [83].

Interestingly, despite The Cancer Genome Atlas (TCGA) data suggesting that DDB2 mRNA expression levels are positively correlated with long-term survival in patients with BC, Kattan and colleagues found that the knockdown of DDB2 could decrease proliferation and colony formation in MCF7 cells. In contrast, DDB2 overexpression promoted cell growth and colony formation in MDA-MB231 cells [43]. Their study showed that DDB2 functions as an oncogene in BC. However, this hypothesis needs to be validated in other breast cancer cell lines.

DDB2 acts as a co-activator in E2F1-mediated transcriptional activation, promoting cell cycle progression, especially during entry into the S phase [43]. E2F1 is a proliferation marker of BC [90]. DDB2 indirectly regulates the expression of critical genes involved in DNA replication and the G1/S transition with E2F1 transcriptional capacities, such as DHFR, cyclin E, and PCNA [18, 91]. In addition, DDB2 stimulates cell growth via the transcriptional repression of MnSOD [80]. DDB2 interacts with the specific DNA sequence AGCCTGCAGCCT in the proximal promoter of SOD2 (superoxide dismutase 2), resulting in the removal of H3 histone acetylation and recruitment of the AP-2α transcription factor. This inhibits the expression of the *SOD2* gene and downregulates the constitutive expression of MnSOD, which in turn causes the accumulation of ROS involved in the activation of various signaling pathways in BC cell growth [80].

In addition, Barbieux et al. used atomic force microscopy (AFM) technology to demonstrate that the DDB2 protein is involved in early BC cell metastasis events by promoting cortical actin cytoskeleton remodeling and inducing changes in adhesion-related gene expression to reduce cell adhesion [92]. Therefore, DDB2 could be used as a novel marker of metastatic progression in BC.

## DDB2 influences sensitivity to chemotherapy and radiotherapy

Given the role of DDB2 in DNA repair, its deletion increases the sensitivity of breast cancer, HNSCC, and other cancer cells to genotoxic therapies such as PARPi (ADP-ribose polymerase inhibitors), the chemotherapeutic drug cisplatin, and radiotherapy [93]. Zhao et al. found that a loss of DDB2 increased the polyubiquitination and proteasome degradation of Rad51, inhibited the homologous recombination repair (HR) pathway, and made TNBC cells more sensitive to DNA damage treatment [94]. It is worth noting that ER-positive breast cancer cells are not sensitive to chemotherapy, which may be related to the ERα-mediated upregulation of DDB2 expression. Recent studies have revealed that ERα induces chemoresistance in BC cells with p53 mutations. ERα upregulates impaired DDB2 transcription by hijacking mutp53, thereby inhibiting the expression of lincRNA-p21 by targeting non-B-DNA structures and promoting subsequent DNA repair and chemoresistance [95]. Inducing lincRNA-p21 and targeting DDB2 will increase chemosensitivity in patients with mutp53 BC.

Chemotherapy resistance has always been a challenging obstacle for patients with cancer to achieve satisfactory therapeutic effects. However, the ability to escape apoptosis caused by the dysregulated expression of apoptotic factors appears to be essential for OC cells to acquire chemoresistance. In contrast to BC, DDB2 deficiency causes OC cells to acquire cisplatin resistance [96]. DDB2 and DDB1 synergistically inhibited Bcl-2 transcription. This process involves DDB2 recognition and binding to the BCP1 site at the 5′-end of the Bcl-2 P1 promoter. DDB1 associates with DDB2 then recruits HDAC1 (histone deacetylase 1) to the P1 core region located 2.2 kb downstream of BCP1 to deacetylate histone H3K9,14, inhibiting Bcl-2 transcription, stimulating the P53-independent apoptosis of cancer cells, and mediating the sensitivity of OC cells to chemotherapy [79].

Radiotherapy plays a crucial role in cancer treatment. However, unexpected radiotherapy resistance is observed in many patients during treatment, resulting in suboptimal treatment outcomes. Although there are several studies on cancer radiation resistance, its potential underlying mechanisms remain unclear given the heterogeneity of tumors and the role of multiple factors in this phenomenon. It has been reported that DDB2 is a potential regulator of radiosensitivity in non-small cell lung cancer (NSCLC) cells. Under ionizing radiation (IR), the level of DDB2 transiently increases and promotes phosphorylation of the G2-blocker mediator Chk1, enhancing the activity of the DNA double-strand break (DSB) homologous recombination repair pathway, inhibiting cancer cell apoptosis, and ultimately leading to radiation resistance in NSCLC [97]. In addition, Sun et al. found that DDB2 overexpression could induce the expression of the anti-apoptotic protein cFLIP (cell membrane-like inhibitory protein) in cisplatin-resistant HeLa cells, partially inhibit TNF-induced apoptosis, and increase the UV resistance in HeLa cells [98]. These results suggest that DDB2 can be used to predict chemoradiotherapy sensitivity.

## Novel discovery: role of DDB2 in other diseases

The hepatitis B virus (HBV) relies on the host DNA repair mechanism to convert virus-relaxed circular DNA (rcDNA) into covalently closed circular DNA (cccDNA) in the nucleus [99]. Marchetti et al. found that UV-DDB, especially the binding activity of DDB2 to DNA and DDB1, and its ubiquitination activity, play an indispensable role in HBV infection [100]. DDB2 must combine with DDB1 to scan the nuclear HBV rcDNA, sense DNA damage, and be ubiquitinated to transfer the rcDNA to the next factor in the cccDNA formation pathway. CccDNA formation was significantly blocked in HepG2-NTCP-DDB2-knockout cells, and the expression of downstream cccDNA markers, such as HBV RNA, HBcAg, and HBeAg, was likewise reduced. DDB2 recruits ATR kinases to UV-damaged DNA sites to initiate the DNA damage response (DDR) involved in forming CM-rcDNA, a hypothetical intermediate precursor for cccDNA formation [5, 101]. Therefore, DDB2 plays a vital role in cccDNA formation and further improves antiviral treatments against hepatitis B.

Qiu et al. found that the DDB2 miRNA target SNP rs1050244 CT/TT genotype was correlated with a reduced risk of hepatocellular carcinoma (HCC) in non-HBV-infected people [102]. It is speculated that the DDB2 SNP rs1050244 may interfere with the targeted interaction of miRNAs (miR-133a and miR197), resulting in the upregulation of DDB2 mRNA expression, thereby reducing an individual’s susceptibility to HCC. However, this result needs to be confirmed through functional experiments.

In addition, by studying the colocalization signals in different brain regions and immune cells expressing quantitative characteristic loci, Tietz et al. found that DDB2 may be the pathogenic gene that causes anti-NMDAR receptor (antiNMDAR) encephalitis [103].

## Concluding remarks and outlook

In this review, we found that DDB2 might target other proteins for degradation and affect many biological processes, including DNA damage repair, DNA replication, and carcinogenesis. As another subunit of the DDB protein complex, DDB1 is a tumor-promoting factor, and its expression is upregulated in pancreatic cancer, liver cancer, and osteosarcoma. Its mechanism may be related to the promotion of cell cycle progression by regulating two key factors, p21 (a CDK2 inhibitor) and CDK2 (cyclin-dependent kinase 2), and the enhancement of EMT by upregulating SNAI1 and ZEB1 [6, 104–106]. However, DDB2 plays a dual role in carcinogenesis by regulating the growth and apoptosis of cancer cells. It also has a broader role than DDB1, since it may play a more important role in the prognosis and predictive biomarkers of cancer. DDB2 levels may also affect the effectiveness of anticancer drugs in cancer treatment and be used as a prognostic factor for the sensitivity of patients with cancer to chemoradiotherapy. Further research in these aspects will broaden our understanding of the multifaceted functions of DDB2 and provide new strategies for treating other human diseases, such as hepatitis. At present, research on DDB2 is not extensive enough, and joint efforts are still needed to explore the broader prospects of this protein.

## Data Availability

Not applicable.
